# Body mass index and obesity-related behaviors in African American church-based networks: A social network analysis

**DOI:** 10.1371/journal.pone.0281145

**Published:** 2023-03-13

**Authors:** Soohyun Nam, Sunyoung Jung, David Vlahov, Carl Latkin, Trace Kershaw, Robin Whittemore

**Affiliations:** 1 School of Nursing, Yale University, Orange, Connecticut, United States of America; 2 College of Nursing, Pusan National University, Pusan, Republic of Korea; 3 School of Public Health, Johns Hopkins University, Baltimore, Maryland, United States of America; 4 School of Public Health, Yale University, New Haven, Connecticut, United States of America; University of Macerata, ITALY

## Abstract

A growing body of research suggests that obesity can be understood as a complex and biobehavioral condition influenced by social relationships ─social networks. Social network analysis allows us to examine how an individual’s network characteristics (e.g., popularity) are associated with obesity and obesity-related behaviors. The objectives of the study were to (a) examine whether network members in African American churches are similar in body mass index (BMI) and obesity-related behaviors (physical activity, eating, alcohol consumption) and (b) examine whether an individual’s network characteristics, such as popularity (i.e., receiving nominations from peers) and expansiveness (i.e., sending nominations to peers) are associated with BMI and obesity-related behaviors. We used a cross-sectional study design and conducted social network analysis using Exponential random graph models with three African American church-based social networks (network A, B, and C, n = 281). There were no significant network members’ similarities on BMI in the three church-based networks. One out of three networks showed similarities in fruit and vegetable consumption (network B), fast food consumption (network C), physical activity, sedentary behaviors, and alcohol consumption (network A). African Americans with a high BMI were more popular, as were individuals with greater fat intake and alcohol consumption. Our findings support the perspective that we need to improve obesity-related behaviors by targeting influential individuals and existing ties and to develop obesity interventions using social networks. The degree to which our findings varied across churches also suggests that the relationship among an individual’s obesity-related behaviors and network characteristics should be understood in the unique social context.

## Introduction

Obesity and obesity-related conditions such as type 2 diabetes, cardiovascular disease, and some types of cancers disproportionately affect African Americans’ health and well-being [1, 2]. Compared to non-Hispanic Whites, African Americans also reported higher rates of physical inactivity and calorie dense food consumption, which are well known obesity-related behaviors [1, 3, 4]. The effects of alcohol consumption on obesity have been mixed and more nuanced depending on amount of alcohol consumption, age, gender and racial groups. However, in recent reviews, alcohol consumption has been linked to obesity and weight gain in cross-sectional and longitudinal studies, and was associated with other obesity-related behaviors such as physical activity [4–6].

Despite substantial public health efforts to reduce obesity, current behavioral interventions to address obesity in African Americans, a high-risk, underserved population have not been successful. A growing body of research has shown that obesity is a complex and biobehavioral condition that can best be understood in a social context going beyond an individual level, and a social (sociocentric) network approach may be a promising method [7, 8]. There is evidence that obesity-related behaviors such as patterns of physical activity, eating, and alcohol consumption may be shared through social networks [7, 9–11]. Behavioral interventions informed by an understanding of social networks associated with obesity-related behaviors may have potential to reduce obesity in African Americans [12].

In social network research, social networks include egocentric networks with an individual at the center −from the perspective of the individual − or sociocentric networks with entire networks within the boundary (i.e., ideally interviewing all connected individuals within the boundary). Egocentric network data are collected from respondents (index persons) about their network members without interviewing those network members. The egocentric network data are analyzed using conventional statistical methods (e.g., regression analysis). In this paper, we will present and discuss social network analyses and findings of the studies that used a sociocentric network approach. Table 1 shows key terms and definitions used in the paper.

**Table 1:** Key terms and definitions used in this paper.

Term	Definition
Egocentric	Network data collected from respondents about their contacts without interviewing those contacts.
Sociocentric (sociometric)	Network data collected from the boundary community.
Actor	A respondent in one of the African American church networks.
Node	An object that may or may not be connected to other objects in a network. In this study, nodes represent respondents who participated in the study (= actor).
Tie	A connection (link) between two nodes.
Density	The number of connections in the networks (ties present divided by number of possible ties)
Ego	The person whose network and behavior are being analyzed.
Alter	A person who is named as a friend by the ego. In other words, an actor who is connected to the ego who may influence the behavior of the ego.
Centrality	Centrality is the extent to which a person inhabits a critical position in the network. Centrality is a node or person-level measure (vs. “centralization” is the extent to which the network is focused on one or a few people and refers to a network-level measure)
Degree	The number of links to and from a person.
In-degree	Number of ties received. This a measure of the number of friendship tie nominations one receives and reflects a dimension of popularity.
Out-degree	The number of ties sent. This is an indicator of the general tendency to send friendship nominations, and reflects the actor’s expansiveness or sociality in a network.
Reciprocity	The tendency to have mutually reciprocated friendships among any two people (i.e., ties to go in both directions: from A to B and B to A).
Transitivity	The tendency to choose a friend of a friend as a friend (i.e., Friends of friends are friends)

Social network analysis provides distinct measures and tools to understand the structure of networks (network-level analysis) and health behaviors of individual network members (individual-level analysis). First, network-level analysis examines structures of networks (e.g., density) and has potential application as a planning, diagnostic and evaluation tool in group or community-based interventions [8]. For example, if the network is sparse (i.e., low density) and not well connected, building networks may be necessary to increase group cohesion and effectively spread the effect of health interventions in the community. Second, while network-level analysis characterizes network structures to understand connectedness among people, and overall properties of the network, individual-level analysis may address unique research questions such as whether individual’s network characteristics (e.g., individual’s position in networks: central, periphery, bridge, isolate) are associated with his/her health outcomes including health behaviors [8].

Physical activity, eating, and alcohol consumption are “social behaviors” that people often share and are influenced by social norms, social learning, and social support—social influence [9, 13]. Through observing others’ behaviors and comparing to one’s own behaviors, people convey social influence by defining social norms about which behaviors are appropriate for a given social environment. Often the most important reference group for an individual is his/her social networks [14]. Members of social networks such as peers or family members learn not only from their own experience but also by modeling or imitating other’s behavior—social learning [14]. Another prominent feature of social network influences is social support. Network members provide one another social support ─ emotional, information, financial and material support ─which is highly associated with health outcomes [14].

Social selection (homophily: ‘birds of a feather flock together’) is another potential social process that may be associated with obesity or obesity-related behaviors [15]. This conception is that people tend to cluster together based on shared outcomes or beliefs. For example, people may select friends based on the similarity (e.g., race) or similar behaviors (e.g., smoking). In a recent systematic review of social network studies, individuals with similar body weight status or weight-related behaviors were more likely to share a network tie (social relationship) than individuals with dissimilar traits [16].

ERGMs are relatively new statistical models for expressing structural properties of social networks and are used to identify whether the particular configuration of ties that occur more or less than would be expected at chance, given the number of nodes and density of the network by generating simulated networks [17]. The most frequently used centrality measure of social networks is degree (Table 1). In-degree counts the number of times a person is nominated by others in the network. In-degree identifies opinion leaders in a network and in friendship networks it indicates popularity [12]. Identifying opinion leaders or popular people is often important to promote behavior change in a group setting. Out-degree is the number of names a person provides in response to a network question (i.e., the number of close friends, the number of sexual partners). Out-degree is a useful indicator for personal attributes such as person’s socialness or sociality and often referred to as expansiveness [12]. Although little is known about how an individual’s popularity or expansiveness is associated with obesity-related behaviors in African American adults, social network studies of health behaviors in other populations have shown the relationship between popularity/expansiveness and health behaviors. In a longitudinal study of 5104 adolescents, those not connected to the rest of the network (neither popular nor sociable, i.e., isolates) were the most likely to use substances [18]. In a review of social networks in dietary behavior in youth, more popular adolescents tended to consume more unhealthy foods [19]. Youth with greater popularity or expansiveness reported engaging in more physical activity than their more isolated counterparts [11, 20].

With a growing interest in social networks and health research, a few studies have been conducted to examine body size norms and weight loss of Black and Hispanic adults in the context of social networks using an egocentric network approach [21, 22]. Also, several reviews have identified gaps in research regarding how social network properties were associated with obesity-related behaviors in adults [23, 24]. Among African Americans, the church has been a central community resource and a key setting for health intervention recruitment and participation [25]. In a recent study of African American church-based social networks, the importance of understanding social network structures for developing group-based health promotion programs at the network-level was demonstrated [26]. To date, however, no published study is available that examines how African American individual’s network characteristics (i.e., individual-level network analysis) are associated with their body mass index (BMI) and obesity-related behaviors using a sociocentric network approach. Individual-level network analysis may provide potential tools to identify and target key players in the networks to enhance the effect of future group- or community-based obesity interventions. Most studies of social networks and health outcomes have reported findings from predominantly white populations, including children.

The purposes of the study were twofold: (a) to examine whether network members in African American churches are similar in BMI and obesity-related behaviors (physical activity, eating, alcohol consumption) and (b) to examine whether an individual’s network characteristics such as popularity and expansiveness (i.e., sociality) are associated with BMI and obesity-related behaviors. Our hypothesis is: (a) network members in African American churches will be similar in their BMI and obesity-related behaviors. Exploratory hypotheses are: (b) popularity or expansiveness will be positively associated with higher BMI, more physical activity, unhealthy eating, and alcohol consumptions.

## Materials and methods

### Design, participants and procedures

We used a cross-sectional study design to examine the network structure and individuals’ network characteristics and their relationships with BMI and obesity-related behaviors within three African American church congregations in New England urban area. Eligibility criteria for participants included the following: (a) men or women over 21 years of age, (b) self-reported Black or African American, and (c) able to speak and read English. We excluded individuals who reported disabilities or acute/terminal conditions (e.g., terminal cancer, dialysis) that affect daily physical activity, or active psychiatric illnesses such as thought disorders. Informed consent was obtained from all individual participants included in the study. Eligible and consenting participants completed self-administered surveys (30–40 minutes) and anthropometric measurements and received a $30 gift card. All study protocols were reviewed by the Yale University Institutional Review Board prior to study implementation.

We collaborated with Yale University Center for Clinical Investigation (National Institute of Health, Clinical and Translational Science Award), “Community Partnership” working group, which comprised local ministers and church leadership groups. Black community leaders from the local chapter of the National Association for the Advancement of Colored People also provided guidance for the recruitment plan. We presented the study purpose, described study-related activities, and discussed effective recruitment strategies in small group meetings in the churches such as bible studies, prayer meetings, and choir group practices. Because church rosters were not available to us, we visited each church every Sunday consecutively for 4–6 months per church to obtain optimally complete sociocentric network data by identifying people who either had already participated or declined to participate. Pastors and the study’s principal investigator announced the study at a Sunday service to initiate recruitment and then every 3–4 weeks to encourage participation. We enrolled and collected data every Sunday and some weekdays at the church from May 2015 to June 2016. The seating capacity for Sunday worship of the three churches (network A, B, & C) was approximately 150, 170, and 230 people. The range of Sunday worship attendance was from 80 to 170 per church including children. Approximately 75% of church congregations from each church participated in the study based on the mean Sunday worship attendance rates (97.3, 126, and 151.3 people).

#### Statement regarding ethical approval

All procedures performed in studies involving human participants were in accordance with the ethical standards of the institutional and/or national research committee and with the 1964 Helsinki declaration and its later amendments or comparable ethical standards.

### Variables and measures

#### Sociodemographic characteristics

Sociodemographic data included age, gender, educational level, and annual household income.

#### Anthropometric characteristics

We weighed each participant on a calibrated digital scale and measured height using a portable stadiometer in a private space at the church. BMI was calculated as weight (kg)/height squared (m^2^). Percent body fat was estimated using the same digital scale that measures foot-to-foot bioelectric impedance. Waist circumference was taken at the narrowest part of the torso, at the end of a normal expiration. Hip circumference was taken around the buttocks in a horizontal plane at the level of maximal extension of the buttocks. The average of three measurements was calculated, and inter- and intra-observer reliability were checked [27].

#### Obesity-related behaviors

Diet behaviors were measured by following validated instruments. The All-Day Screener of the Eating at America’s Table Study was used to assess *daily fruit and vegetable intake* [28]. The All-day Screener consisted of 19 items on the frequency and portion size of fruits and vegetables consumed over the past month (“Never = 0” to “≥5 items/day = 9”). The portion size was rated on a 4-point scale depending on each fruit or vegetable type (e.g., from < 3/4 cup [= 1] to > 2 cups [= 4]). The daily fruit and vegetable intake were calculated by the scoring method of the National Cancer Institute (NCI) using the times/day and cup equivalents of the 2005 MyPyramid for each portion size category; a higher score indicates more daily fruit and vegetable intake. *Percentage energy from fat intake* was measured by the 13-item NCI Fat Screener [29]. Participants chose the frequency of a particular food or food group, ranging from 1 (never) to 8 (≥2 /day). The mean fat daily intake was estimated by a scoring algorithm, using the values for the age-gender specific portion sizes and the regression coefficients developed by NCI; a higher score indicates greater percentage energy from fat. *Fast food consumption* was assessed by asking how many times food was purchased at a restaurant where food was ordered at a counter or at a drive-through window in the past month (from never or rarely = 1 to 3 ≥/day = 9), with a higher score indicating higher fast food consumption.

The 8-item Paffenbarger Physical Activity Questionnaire (PPAQ) was used to capture habitual physical activities, structured exercise, and sedentary activities [30]. A *physical activity index* (PAI) provides an estimate of weekly energy expenditure by taking the sum across all items after multiplying the kcal/day score for each activity [31]; a higher score indicates more energy expenditure. *Total weekly activity* (TWA) was measured by the number of hours in a typical weekday and weekend day subjects spent sleeping, engaged in quiet sitting activity, in light activity, moderate activity, or vigorous activity. Then a metabolic equivalents (MET) score was assigned by the intensity of each activity: vigorous = 7 MET, moderate = 4.5 MET, light = 3 MET, sitting = 1 MET, and sleeping = 0.8 MET. Thus, the TWA scale is expressed in units of MET*h per week. Overall scores for TWA were calculated by taking a sum across all items of weekday and weekend; a higher score indicates more total weekly activities. *Sedentary activity* was measured by the number of hours spent on eating, reading, desk work, watching TV/movies, listening to a radio, and playing video/computer games in a typical weekday and weekend. An overall score for sedentary activity was calculated by taking a mean of the weekday and weekend scores. The validity and reliability of the PPAQ has been supported in many studies [31, 32].

*Alcohol consumption* was asked by “How many days did you drink more than a glass of alcohol during the last month?” (A glass of alcoholic beverage means one can of beer, a glass of wine, a glass of cocktail, etc) and “How often do you have five or more drinks on one occasion?”

#### Social networks

A name generator survey was used to elicit actors’ social networks within the church [12]. A name generator survey asked about actors’ network members who provided emotional, financial, and informational support within the church; network members with whom the individual gets together to socialize or have fun doing things such as shopping, going to the movies or clubs, or just hanging out; network members who the individual sought advice from; type and frequency of shared activities per week or month. Participants were then asked to list the names and relationships of their network members within the church. At the end of the name generator survey, we provided an example table with five rows as a guide. However, if participants wanted to report more or less than five people (their network members), they were instructed to list as appropriate. Based on the name generator answers, an N by N adjacency matrix for each church (= each network) was created, where N is the number of participants in the network. If participant *i* named participant *j* as a network member, then the *i*,*j* entry in the matrix was a “one”, and all other entries were “zero.” Thus, each row of the matrix corresponds to a particular participant *i*, called an ‘‘ego,” and each ego is surrounded by his or her local ‘‘alters”: other actors in the network with their own attributes, network characteristics, and behaviors, indexed by the subscript *j*, corresponding to the columns in the adjacency matrix.

### Statistical analysis

Descriptive statistics were conducted to summarize sample characteristics and study variables. Differences among participants of the three churches regarding demographics, BMI, diet behaviors, physical activity, and alcohol consumption were examined with one-way analysis of variance (ANOVA) for continuous variables and with χ^2^ tests for categorical variables. For all analyses, .05 was used as the significance level (two-tailed). To determine whether there was the propensity of an individual’s attributes (i.e., BMI, obesity-related behaviors) associated with the formation of network ties, we conducted ERGMs with Markov Chain Monte Carlo Maximum Likelihood estimates using PNet software, based on a fixed number of nodes and graph density [33]. By fixing the graph density, the number of arcs/edges will not change during estimation and facilitate the convergence for parameter estimation [17, 34].

#### ERGMs

ERGM parameters represent a range of different tie configuration; for example, reciprocity (the extent to which ties are reciprocated) and transitivity (shared friendship; ‘friends of my friends are also my friends’)—each of which relates to specific structural processes between network ties and node level attributes [17]. That is, the formation of ties—the network structure—is assumed to be based on a structural (‘endogenous’) process such as reciprocity or transitivity, as well as on an ‘exogenous’ process (node [actor or individual] level attributes), including social selection [33]. In an ERGM, networks are treated as an endogenous process and actor attributes are treated as exogenous or explanatory variables that affect the presence of social ties [33]. Therefore, ERGMs can be used to describe a network structure and determine if individual attributes (node level characteristics) are associated with network structural properties [12]. The statistical models based on stochasticity assumptions estimate which effects, such as network structural properties and health behaviors, significantly explain the network structure to determine whether particular configurations of ties occur more or less than expected by chance, given the fixed number of nodes and density of the network and given other effects in the network model. A positive parameter suggests the effect is more prevalent and a negative parameter indicates that the effect is less prevalent than chance, given the other effects in the model [34].

**Fig 1:**
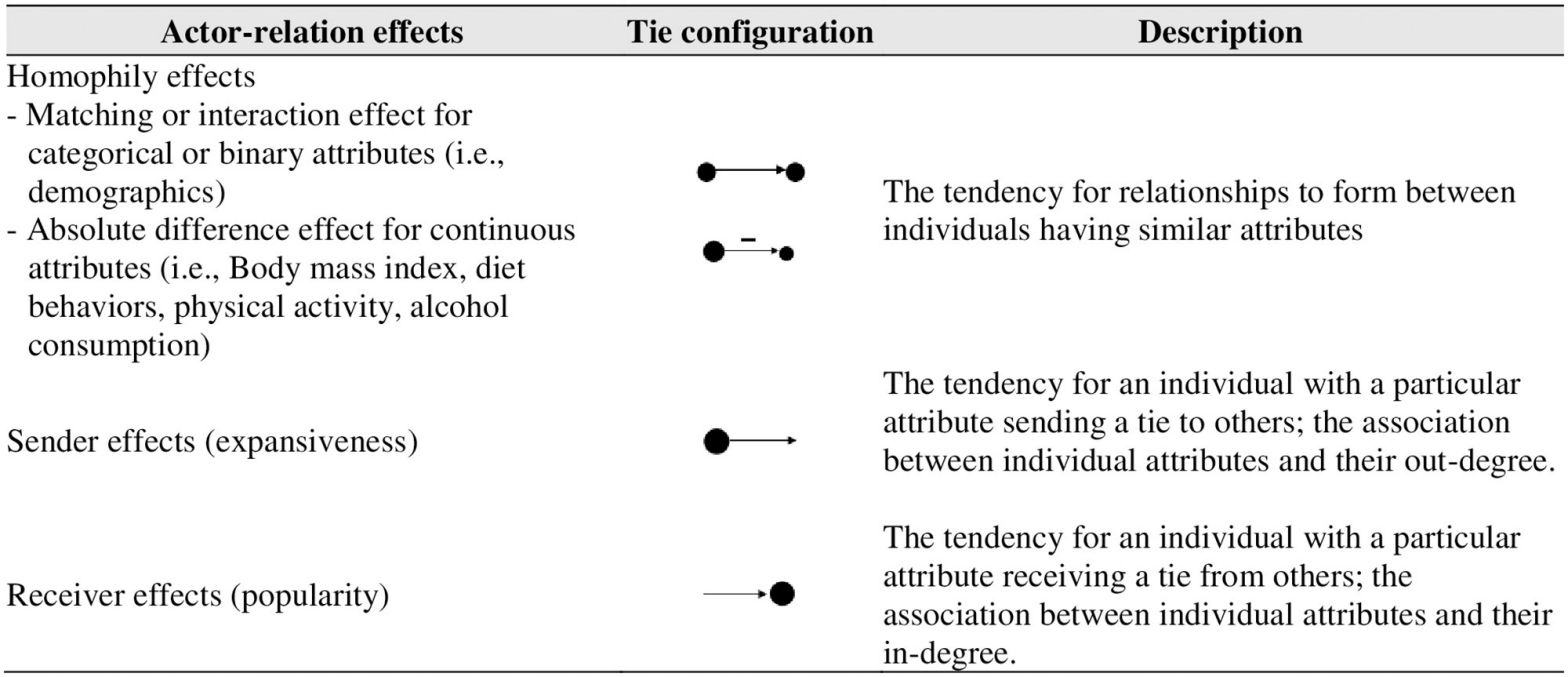
Tie configurations and description of individual-relation effects. Note: Based on Lusher, Koskinen, & Robins [33]; Snijders, Pattison, & Robins [46].

“Actor-relation effects,” which refer to “the association of a particular attribute with a social relationship tie,” were analyzed with parameters of three types of effects: homophily, sender, and receiver effect [33]. Fig 1 shows the configurations and descriptions for the actor-relation effects used in this study [33].

To assess the homophily effect by demographic characteristics of linked ties, a matching (for categorical attributes) or interaction (for binary attributes) parameter was included in the model specification. The homophily effect of BMI and obesity-related behaviors were estimated by an absolute difference for an attribute between individuals who shared a directed tie; a significant negative estimate indicates that linked network members have a similarity on BMI or each obesity-related behavior (i.e., have less of a difference than expected by chance). The parameter for sender effects, which represents an association between out-degree (the number of social contacts named by an individual) and each obesity-related behavior were included for each continuous attribute; a significant positive estimate indicates that a high value on this attribute is associated with sending more ties (i.e., nominating a great number of network members). In other words, if the parameter estimate is positive and large, the parameter associated with expansiveness are more probable in the model. Similarly, receiver effects indicating an association between each continuous attribute and in-degree were also estimated; a significant positive value indicating a high value on this attribute is associated with receiving more ties. We also computed odds ratios (OR) for the ERGM parameter estimates to help in interpreting the magnitudes of associations relative to the other effects included in each model [34, 35].

In all the actor-relation effect models, we controlled the structural network parameters (i.e., reciprocity, popularity, and transitivity) [33] and significant actor-relation effects of demographics (i.e., age and gender) were also controlled.

The standard errors test the significance of the result by calculating a t-statistic. A parameter estimate greater than twice its standard error was considered statistically significant (commonly expressed as p<0.05) [33]. Convergence of the estimation algorithm was assessed by a t-ratio (parameter observation-sample mean/standard error) [17]. Goodness-of-fit (GOF) was assessed by examining simulations of the observed networks generated from the estimated parameters using PNet [33]. The GOF t-ratios for the estimated parameters (e.g., reciprocity, transitivity) should be smaller than 0.1 in absolute value. For statistics of the graph features that were not specifically included in our models, the GOF statistics with a t-ratio smaller than the absolute value of 2.0 are considered as a reasonable model fit [33].

## Results

### Sociodemographic characteristics and obesity-related behaviors

Table 2 shows the sociodemographic characteristics and obesity-related behaviors from three church-based social networks (Network A, B, & C) with 281 African American men and women. The sample included 100% self-identified African American and 32.4% were currently married. About 89% were either overweight (BMI 25–29.99 kg/m^2^) or obese (BMI ≥30 kg/m^2^). There was no significant difference in obesity-related behaviors among the three churches. However, significant differences were found in gender, income, mean BMI, and mean body fat percentage among the three churches.

**Table 2:** Description of sociodemographic characteristics, body mass index, and obesity-risk behaviors (n = 281).

Variables	Mean (SD) or N (%)				F or χ^2^	p
	Total (n = 281)	Network A (n = 113)	Network B (n = 95)	Network C (n = 73)		
Age^a^ (years)	52.8 (14.8)	53.7 (16.8)	52.9 (12.2)	51.3 (14.8)	0.58	.56
Gender^b^					9.17	.01*
Woman	216 (76.9)	94 (83.2)	75 (78.9)	47 (64.4)		
Education level^b^					7.81	.10
High school graduate or below	157 (55.9)	59 (52.2)	51 (53.7)	47 (64.4)		
College graduate	62 (22.1)	21 (18.6)	25 (26.3)	16 (21.9)		
Graduate school or higher	62 (22.1)	33 (29.2)	19 (20.0)	10 (13.7)		
Annual household income^b^					15.07	.02*
0-$39,999	108 (38.4)	35 (31.0)	38 (40.0)	35 (47.9)		
$40,000-$79,999	76 (27.0)	37 (32.7)	24 (25.3)	15 (20.5)		
$80,000 or higher	62 (22.1)	33 (29.2)	18 (18.9)	11 (15.1)		
Refused to answer	35 (12.5)	8 (7.1)	15 (15.8)	12 (16.4)		
Anthropometrics characteristics						
BMI (kg/m^2^) ^a^	32.0 (7.0)	32.3 (7.2)	33.1 (7.5)	30.0 (5.5)	4.36	.01*
<25	32 (11.4)	9 (8.0)	11 (11.6)	12 (16.4)		
25–29	76 (27.0)	30 (26.5)	21 (22.1)	25 (34.2)		
30–39	136 (48.4)	60 (53.1)	44 (46.3)	32 (43.8)		
40 or higher	37 (13.2)	14 (12.4)	19 (20.0)	4 (5.5)		
Waist-hip ratio ^a^	0.9 (0.1)	0.9 (0.1)	0.9 (0.1)	0.9 (0.1)	0.95	.39
Body fat (%)^a^	40.5 (10.2)	41.1 (8.7)	42.4 (10.2)	37.1 (11.5)	6.32	.00*
Diet						
Fruit/vegetable intake (servings/day) ^a^	3.3 (4.8)	2.8 (2.3)	4.2 (7.1)	2.7 (3.5)	2.87	.06
% energy from fat ^a^	33.4 (5.0)	33.3 (4.4)	33.7 (6.6)	33.2 (3.3)	0.21	.81
Fast-food consumption ^b^					2.75	.07
2–3 times or less/month	175 (62.3)	66 (58.4)	67 (70.5)	42 (57.6)		
1 or more times/week	89 (31.7)	35 (31.0)	25 (26.3)	29 (39.7)		
1 or more times/day	17 (6.0)	12 (10.6)	3 (3.2)	2 (2.7)		
Physical activity						
PAI (kcal/week) ^a^	11,988.9 (2,917.2)	11,593.8 (1,734.3)	2,255.6 (3,299.3)	22,253.5 (3,714.0)	1.74	.18
TWA (MET*h/week) ^a^	406.6 (95.7)	402.4 (92.0)	402.7 (101.9	418.2 (93.3)	0.72	.49
Sedentary activity (h/week) ^a^	41.1 (17.9)	41.0 (19.2)	41.6 (17.5)	40.8 (16.6)	0.44	.96
Alcohol (days/month) ^a^	2.3 (5.1)	2.5 (5.7)	1.8 (4.6)	2.6 (4.8)	0.67	.52

Note: Network A (= Church A),

*P<0.05 significance;

Body Mass Index (BMI); Physical Activity Index (PAI) is a weekly energy expenditure calculating by walking, stair climbing, and sports or recreational activities. Total Weekly Activity (TWA); Metabolic Equivalents (MET); Sedentary activity include eating, reading, desk work, watching TV, listening to radio, etc.

^a^, continuous variables;

^b^, nominal variables.

### Network structural properties in African American church-based social networks

**Fig 2:**
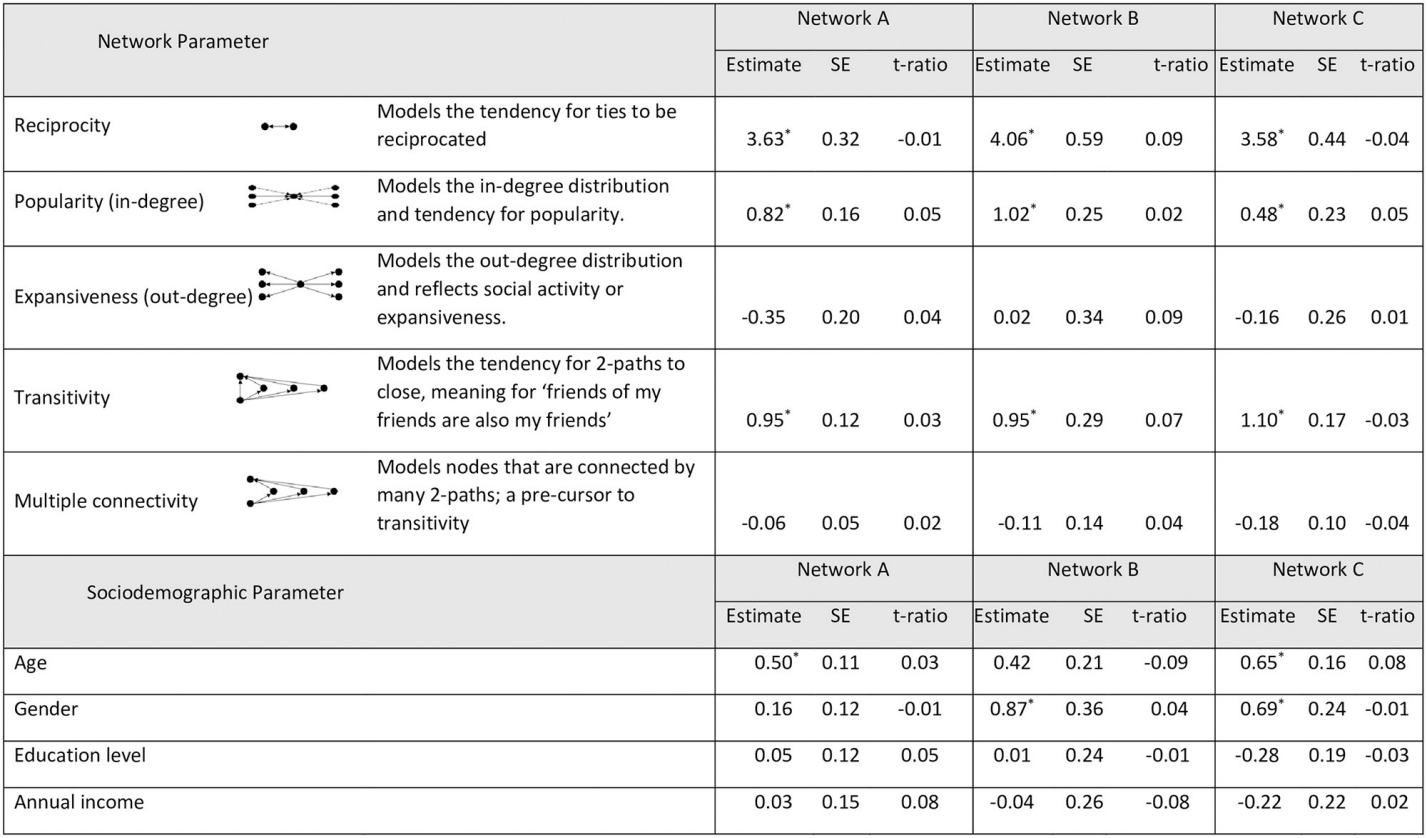
Effects of network structures and sociodemographic attributes on the church-based social networks by exponential random graph models (n = 281). Note: * Significant effect (i.e., parameter estimate is greater than two times the standard error in absolute value). SE; standard error. The parameter is the weight applied to the statistics (just as in a logistic regression with predictor variables and regression coefficient). An Exponential Random Graph Model (ERGM) assigns a probability to a graph by a sum of statistics weighed by parameters. For example, if the estimate of reciprocity parameter is large and positive, then graphs with many reciprocities are more probable in the graph distribution for that model. However, if the estimate of reciprocity parameter is large and negative, then graphs with fewer reciprocity become more probable under the model. Convergence of the estimation algorithm assessed by a t-ratio (parameter observation-sample mean/standard error): the absolute value should be < 0.1.

The sizes of three networks were 113, 95 and 73. Density (0.01, 0.01, 0.02), average degree (1.40, 0.60, 1.16), reciprocity (0.21, 0.14, 0.23), transitivity (0.08, 0.08, 0.14), clustering coefficient (0.21, 0.17, 0.20) and centralization (0.13, 0.05, 0.05) were similar among the three churches. The detailed network level findings have been published elsewhere [26]. Overall, 61% of participants among the three churches reported that their network members were friends; 3% were household members; and 17% were non-household family members such as siblings and relatives. Significant reciprocity effects were shown in all three networks, indicating that the ties tended to be reciprocated between dyad. There were significant popularity effects in all three networks, meaning that nodes with high in-degrees (actors who received many nominations) tended to exist. All three networks also had significant transitivity effect, indicating that there was a tendency for ‘friends of my friends are also my friends’ (shared friendship). The tendencies that social networks form within similar age and gender groups after controlling for the aforementioned, significant structural effects, such as reciprocity, popularity, and transitivity were shown Fig 2.

### Effects of absolute difference, sender, and receiver on BMI and obesity-related behaviors

We controlled for the structural (‘endogenous’) effects, age, and gender found in the observed networks from the previous analyses (Fig 2). The GOF statistics for each network indicated that the models had a good fit with the t-ratios of an absolute value of less than 0.1 for the estimated parameters (the selected GOF details were provided in the supplementary material).

Table 3 shows estimates of actor-relation effects (absolute difference, sender, receiver) on BMI and obesity-related behaviors, controlling for significant structural effects (i.e., reciprocity, popularity, transitivity) and significant demographic homophily effects (i.e., age, gender).

**Table 3:** The actor-relation effects on body mass index and obesity-related behaviors (with odds ratio and 95% CI).

Actor-relation effects	Network A						Network B						Network C					
	Estimate	SE	t-ratio	Odds ratio	95% CI		Estimate	SE	t-ratio	Odds ratio	95% CI		Estimate	SE	t-ratio	Odds ratio	95% CI	
					Lower	Upper					Lower	Upper					Lower	Upper
**Absolute difference effect**																		
Body mass index	0.05	0.06	0.09	1.05	0.94	1.18	-0.06	0.14	-0.01	0.94	0.72	1.24	-0.07	0.12	-0.03	0.93	0.74	1.18
Diet behaviors																		
Fruit/vegetable intake	-0.18	0.12	-0.07	0.84	0.66	1.06	-1.14*	0.47	-0.04	0.32	0.13	0.80	-0.16	0.11	-0.01	0.85	0.69	1.06
% energy from fat	-0.05	0.07	-0.05	0.95	0.83	1.09	-0.17	0.17	0	0.84	0.61	1.18	0	0.09	0.07	1.00	0.84	1.19
Fast-food consumption	-0.11	0.07	0	0.90	0.78	1.03	0.13	0.1	-0.03	1.14	0.94	1.39	-0.25*	0.12	0	0.78	0.62	0.99
Physical activity																		
PAI (physical activity index)	-0.19*	0.09	0.05	0.83	0.69	0.99	0.26	0.31	-0.01	1.30	0.71	2.38	-0.08	0.18	0.02	0.92	0.65	1.31
TWA (total weekly activity)	-0.08	0.07	0.04	0.92	0.81	1.06	0	0.13	-0.02	1.00	0.78	1.29	-0.12	0.12	0.06	0.89	0.70	1.12
Sedentary activity	-0.17*	0.08	0.04	0.84	0.72	0.99	-0.09	0.15	-0.03	0.91	0.68	1.23	-0.1	0.13	-0.02	0.91	0.70	1.17
Alcohol consumption	-0.32*	0.09	-0.02	0.73	0.61	0.87	0.23	0.51	0.02	1.26	0.46	3.42	-0.39*	0.17	0.07	0.68	0.49	0.95
**Sender effect**																		
Body mass index	0.07	0.08	0.05	1.07	0.92	1.26	0.06	0.14	0.01	1.06	0.81	1.40	-0.17	0.12	0.02	0.84	0.67	1.07
Diet behaviors																		
Fruit/vegetable intake	0.15	0.11	-0.05	1.16	0.94	1.44	1.26*	0.45	-0.05	3.53	1.46	8.52	0.30*	0.11	-0.02	1.35	1.09	1.68
% energy from fat	-0.09	0.08	-0.07	0.91	0.78	1.07	0.19	0.17	0.01	1.21	0.87	1.69	-0.07	0.12	-0.06	0.93	0.74	1.18
Fast-food consumption	0.05	0.09	0	1.05	0.88	1.25	0.04	0.13	-0.08	1.04	0.81	1.34	0.21	0.14	0.03	1.23	0.94	1.62
Physical activity																		
PAI (physical activity index)	0.1	0.08	0.03	1.11	0.95	1.29	-0.64*	0.3	0.02	0.53	0.29	0.95	0.22	0.18	0.03	1.25	0.88	1.77
TWA (total weekly activity)	-0.18*	0.09	0.03	0.84	0.70	0.99	-0.04	0.13	-0.06	0.96	0.75	1.24	0.24	0.13	0	1.27	0.99	1.64
Sedentary activity	0.24*	0.09	-0.02	1.27	1.07	1.52	-0.21	0.16	0.01	0.81	0.59	1.11	0.02	0.13	-0.03	1.02	0.79	1.32
Alcohol consumption	0.21*	0.09	-0.06	1.23	1.03	1.47	-0.11	0.5	0.03	0.90	0.34	2.39	0.18	0.16	0.08	1.20	0.88	1.64
**Receiver effect**																		
Body mass index	-0.09	0.07	0.04	0.91	0.80	1.05	0.26*	0.12	0.01	1.30	1.03	1.64	0.09	0.11	0	1.09	0.88	1.36
Diet behaviors																		
Fruit/vegetable intake	-0.15	0.09	0	0.86	0.72	1.03	-0.49	0.4	-0.03	0.61	0.28	1.34	0.02	0.1	-0.05	1.02	0.84	1.24
% energy from fat	0.14*	0.07	-0.05	1.15	1.10	1.32	-0.05	0.14	-0.06	0.95	0.72	1.25	0	0.12	-0.02	1.00	0.79	1.27
Fast-food consumption	0.07	0.07	0.02	1.07	0.94	1.23	-0.11	0.1	-0.05	0.90	0.74	1.09	-0.09	0.13	0.01	0.91	0.71	1.18
Physical activity																		
PAI (physical activity index)	0.06	0.07	0.04	1.06	0.93	1.22	-0.09	0.29	-0.02	0.91	0.52	1.61	-0.06	0.16	0.02	0.94	0.69	1.29
TWA (total weekly activity)	0.06	0.07	0	1.06	0.93	1.22	0.02	0.11	-0.02	1.02	0.82	1.27	-0.14	0.12	-0.01	0.87	0.69	1.10
Sedentary activity	-0.1	0.07	0.09	0.91	0.79	1.04	-0.08	0.13	-0.02	0.92	0.72	1.19	-0.15	0.12	0.01	0.86	0.68	1.09
Alcohol consumption	0.19*	0.08	-0.06	1.21	1.03	1.42	-0.58	0.47	-0.04	0.56	0.22	1.41	0.17	0.15	0.09	1.19	0.88	1.59

Note:

* Significant effect (p<0.05).

A t-ratio is calculated by (parameter observation-mean sample)/standard error. A t-ratio of an absolute value of less than 0.1 indicates good convergence. The individual-relation effects on age and gender were controlled. Physical Activity Index (PAI) is a weekly energy expenditure calculating by walking, stair climbing, and sports or recreational activities. Total Weekly Activity (TWA); Metabolic Equivalents (MET); Sedentary activity include eating, reading, desk work, watching TV, listening to radio, etc.

Our hypothesis that there would be similarities on BMI and obesity-related behaviors in African American church-based networks was tested by network parameters for absolute difference effects. Significant negative estimates in absolute differences (denoted by an asterisk [*] in Table 3) indicate that there were similarities on obesity-related behaviors. There were no significant BMI similarities among network members in all three churches. In Network B and Network C, network members tended to be alike on some of their diet behaviors; however, in Network A, there was no evidence that network members were alike on diet behaviors. Instead, in Network A, network members were found to engage in similar amounts of physical activity and sedentary activities. Similarities on alcohol consumption among network members were found in Network A (OR = 0.73, 95% CI [0.61,0.87]) and C (OR = 0.68, 95% CI [0.49,0.95]), indicating that social relationships were more likely to form when they had similar alcohol consumption status.

For sender effects (expansiveness) on an individual’s obesity-related behaviors, both Network B (OR = 3.53, 95% CI [1.46,8.52]) and C (OR = 1.35, 95% CI [1.09,1.68]) showed that individuals with higher fruit/vegetable intake were more likely to nominate a larger number of network members. Also, for Network A (OR = 0.84, 95% CI [0.70,0.99]) and B (OR = 0.53, 95% CI [0.29,0.95]), the negative and significant sender effects for physical activity indicated that individuals who were less engaged in physical activity tended to send more ties. In Network A, individuals with more sedentary behaviors (OR = 1.27, 95% CI [1.07,1.52]) and higher alcohol consumptions (OR = 1.23, 95% CI [1.03,1.47]) tended to send more ties.

We examined receiver effects (popularity) on an individual’s BMI and obesity-related behaviors. In Network A, individuals with a high percentage of energy from fat intake (OR = 1.15, 95% CI [1.10,1.32]) and alcohol consumption (OR = 1.21, 95% CI [1.03,1.42]) tended to be popular. For Network B, the positive and significant receiver effect for BMI indicated that individuals with higher BMI tended to receive more ties (popularity) (OR = 1.30, 95% CI [1.03,1.64]). However, the receiver effect for BMI was not found in Network A and C. Also, there was no receiver effect on physical activity and sedentary behaviors in all three networks.

## Discussion

Substantial research of social and behavioral factors and health has focused on dyadic ties and used an egocentric network approach, obtaining data from the index person’s perception [36]. While these studies are valuable, a growing number of studies also suggest that understanding the role of social connections on health behaviors needs to take into account the complex structures of the social relationship ties beyond the examination of dyads and perceptions of index individuals in small groups [24, 37]. We conducted sociocentric networks analyses to examine whether social connections among African Americans were associated with similar BMI and health behaviors in church-based networks. To date, no published research has examined similarities on BMI and obesity-related behaviors among African Americans in church settings, the central hub of social life and community-based health research for African Americans [25].

In our African American church-based social networks, we did not find similarities in BMI among network members. African American adults in church-based networks were found to be similar in some obesity-related behaviors. One out of the three church-based networks showed similarities in fruit and vegetable consumption, fast food consumption, physical activity, and sedentary behaviors. Two of our three church-based networks showed that network members were alike in their alcohol consumption. To date, findings of homophily effect on BMI, health beliefs, and health behavior in social network studies are mixed by types (e.g., sorority, school) and sizes of networks and populations (e.g., race, gender, age) [38–40].

As shown in another study that compared multiple social networks within the study [41], our observed social networks were different from one another. This suggests that it cannot be taken for granted that adult social networks are based around universal behavioral similarities. The varied findings from the small number of networks sampled in our study should be interpreted in the social context, including church level differences in health-related social norms and church membership characteristics. All three churches were African-American Methodist denominations in New England area. Also, these three churches had community outreach groups within the congregation for health promotion. Although we controlled for significant individual’s attributes such as age and gender that may influence network tie formation within the network, there were significant differences in gender, income, mean BMI, and mean body fat across the three churches that may have influenced the degree to which our findings varied across the churches. With our currently available data, the relationship among behavioral homophily, social-environmental influences such as social inequalities (e.g., income, education), and network tie formation cannot be answered [42]. More information on social inequalities within the church and participants’ perceptions about how they build social relationships and adopt health behaviors is needed. The findings across our observed networks warrant mixed methods studies integrating qualitative perspectives. Also, findings suggest that future group-based obesity interventions—where social interaction among participants may change group dynamics and the effect of interventions—may need to be modified to address the local social circumstances.

Social norms about what is socially acceptable influence an individual’s health behaviors within a social network [12]. Although a single observation of social networks with our cross-sectional study design may not disentangle the mechanisms of social processes—social influence and social selection, some findings on BMI or obesity-related behaviors may reflect social cultural norms around body image, food (e.g., soul food) and health beliefs in the African American community [43, 44]. Our participants, African men and women with a high BMI, received popularity in one network. Participants with a high percentage of fat intake or alcohol consumptions were the most popular in one network. While heterogeneity within African American culture is also recognized [45], some of our findings are consistent with salient social norms and beliefs about large body size, social pressure around physical activity, and eating among African Americans [43, 44]. Future longitudinal studies are warranted to understand the underlying social processes that may be unique to local networks and culture in African Americans.

The current study benefited from methodological strengths, including objectively measured weight and height for BMI and comparing multiple church-based social networks using a sociocentric network approach. The study, however, is not without limitations. Limitations of this study, as shown in other social networks studies, are whether localized social process and network structures from observed networks are sufficient to explain global network properties. It may be difficult to investigate such questions without a case of global model resulting from combinations of many small-scale structures [17]. We investigated church-based social networks because churches are often considered to be hubs for providing culturally tailored group interventions and community-based health programs for African Americans [25]. Working with the African American community partners and church leadership groups, we respected their confidentiality concerns using rosters of congregations. Using a free-recall name generator rather than a roster method, which helps as a memory aid in nominating network members and for setting the boundary of networks, could have influenced our findings. Particularly, low average degree observed among the church networks may have been affected by this methodological limitation using a name generator. The small proportion of household family members (3%) and various non-household familial relationships connected by blood vs. marriage made it difficult to analyze with a dyad-level variable to further explore genetic and environmental effects on obesity and obesity-related behaviors. The high prevalence of overweight and obesity among our participants, which reflect the national prevalence of overweight/obesity in African Americans, may not have had variability in BMI and influenced our findings on the relationships with BMI compared to social networks in other populations. We used self-reported, obesity-related behaviors; some of the health behaviors may be over- or underestimated resulting from social desirability [37].

Longitudinal research is also needed to tease apart network dynamics and social influence. Our findings may be influenced by both social selection where individuals adopt behaviors that are similar to those of their network members and other processes of social influence such as social norms, and social support. In any event, our findings support that we need to improve obesity-related behaviors and social norms around obesity by harnessing influential individuals and existing ties (e.g., reciprocated ties) and developing obesity programs with an understanding of social networks [45].

Based on substantial variations found in studies of social networks and health including the current study, it is also important to note that social context such as types, functions and structures of networks is a fundamental precursor to any observed relationship between social relational ties and health behaviors [42]. Studying social contexts within networks using mixed methods research and developing measures of social context relevant to social network and behavioral theories would facilitate future research to improve understanding of social networks and health.

In conclusion, emerging studies continue to demonstrate the notable implications of social networks on health. Our study is the first study that applied ERGMs to examine BMI and obesity-related behaviors among African Americans in church-based networks. The degree to which our findings varied across churches also suggests that the relationship among an individual’s obesity-related behaviors and social network characteristics should be understood in the unique social context and that the development of future obesity intervention may need to address local network circumstances.

## Supporting information

S1 Table The goodness-of-fit (GOF) statistics of each network. (DOCX)

S1 Dataset (XLSX)
