# Supplementary material for: Body mass index and obesity-related behaviors in African American church-based networks: A social network analysis
Source: PLoS One. 2023 Mar 13;18(3):e0281145. doi: 10.1371/journal.pone.0281145 (PMC10010537; doi:10.1371/journal.pone.0281145)
Supplement: S1 Table — (DOCX) [file pone.0281145.s001.docx]

**Supplement 1.** The goodness-of-fit (GOF) statistics of each network

| Network | Category | BMI | Fruit  /vegetable intake | % energy from fat | Fast-food consumption | PAI | TWA | Sedentary activity | Alcohol consumption |
| --- | --- | --- | --- | --- | --- | --- | --- | --- | --- |
| A | Reciprocity | -0.02 | 0.01 | -0.08 | -0.08 | 0.04 | -0.02 | 0.03 | 0.07 |
|  | Popularity | -0.05 | 0.04 | -0.12 | -0.08 | 0.09 | -0.05 | 0.03 | 0.06 |
|  | Transitivity | -0.03 | -0.01 | -0.11 | -0.07 | 0.12 | -0.08 | 0.01 | 0.08 |
|  | Absolute difference | -0.03 | -0.03 | 0.01 | -0.03 | 0.07 | 0.09 | 0.06 | 0.02 |
|  | Sender | -0.02 | -0.06 | -0.05 | -0.07 | 0.00 | 0.10 | 0.04 | -0.03 |
|  | Receiver | -0.05 | 0.04 | -0.06 | -0.07 | 0.02 | 0.10 | 0.01 | -0.03 |
|  | Age | 0.04 | 0.03 | -0.02 | -0.12 | 0.09 | -0.09 | 0.04 | 0.07 |
|  | Gender | -0.01 | -0.02 | -0.04 | 0.04 | -0.03 | 0.02 | 0.05 | 0.00 |
|  | 2-in-star | 1.22 | 1.17 | 0.97 | 1.04 | 1.38 | 0.96 | 1.32 | 1.29 |
|  | 2-out-star | -1.42 | -1.31 | -1.39 | -1.38 | -1.37 | -1.50 | -1.47 | -1.38 |
|  | Isolates | -0.61 | -0.50 | -0.59 | -0.58 | -0.50 | -0.68 | -0.57 | -0.50 |
|  | AoutS(2.00) | -1.34 | -1.24 | -1.33 | -1.30 | 0.12 | -1.48 | -1.43 | -1.29 |
|  | A2p-T | -0.94 | -0.86 | -0.93 | -0.98 | -0.86 | -1.05 | -0.88 | -1.69 |
|  | Standard deviation of in-degree distribution | 1.22 | 1.19 | 1.00 | 1.06 | 1.37 | 0.99 | 1.31 | 1.30 |
|  | Skew in-degree distribution | 1.91 | 1.80 | 1.55 | 1.64 | 2.12 | 1.61 | 2.08 | 1.99 |
|  | Standard deviation of out-degree distribution | -1.55 | -1.45 | -1.55 | -1.53 | -1.51 | -1.69 | -1.63 | -1.53 |
|  | Skew out-degree distribution | -1.99 | -1.95 | -2.06 | -1.99 | -1.89 | -2.17 | -2.03 | -1.96 |
|  | Correlation between the in-degree and out-degree distribution | -1.16 | -1.06 | -1.10 | -1.22 | -1.08 | -1.25 | -0.96 | -1.11 |
|  | Global Clustering Cto | 0.80 | 0.75 | 0.63 | 0.71 | 0.99 | 0.66 | 0.89 | 0.89 |
|  | Global Clustering Cti | -0.68 | -0.66 | -0.75 | -0.68 | -0.59 | -0.78 | -0.63 | -0.63 |
|  | Global Clustering Ctm | 0.33 | 0.32 | 0.20 | 0.30 | 0.52 | 0.17 | 0.35 | 0.43 |
|  | Global Clustering Ccm | -0.53 | -0.51 | -0.62 | -0.54 | -0.44 | -0.63 | -0.47 | -0.47 |
|  | Global Clustering AKC-T | 0.69 | 0.67 | 0.56 | 0.68 | 0.90 | 0.55 | 0.70 | 0.80 |
|  | Global Clustering AKC-D | 1.34 | 1.30 | 1.17 | 1.25 | 1.55 | 1.24 | 1.44 | 1.42 |
|  | Global Clustering AKC-U | -0.58 | -0.57 | -0.66 | -0.59 | -0.49 | -0.69 | -0.54 | -0.53 |
|  | Global Clustering AKC-C | -0.33 | -0.32 | -0.43 | -0.35 | -0.23 | -0.43 | -0.28 | -0.28 |
| B | Reciprocity | -0.12 | -0.08 | -0.09 | -0.05 | -0.07 | 0.04 | 0.04 | -0.03 |
|  | Popularity | -0.09 | -0.09 | -0.07 | -0.05 | -0.01 | 0.01 | 0.04 | -0.04 |
|  | Transitivity | -0.10 | -0.04 | -0.07 | -0.06 | -0.03 | -0.02 | 0.03 | -0.08 |
|  | Absolute difference | -0.05 | 0.03 | 0.05 | -0.07 | 0.02 | 0.09 | 0.04 | 0.00 |
|  | Sender | -0.09 | 0.01 | 0.09 | -0.02 | -0.03 | 0.02 | -0.02 | 0.01 |
|  | Receiver | -0.07 | -0.06 | 0.06 | 0.02 | 0.01 | 0.04 | 0.03 | -0.09 |
|  | Age | -0.06 | -0.09 | -0.08 | -0.10 | -0.07 | -0.02 | 0.02 | -0.04 |
|  | Gender | -0.02 | -0.07 | -0.01 | -0.01 | -0.01 | -0.02 | -0.04 | -0.02 |
|  | 2-in-star | -0.39 | -0.42 | -0.32 | -0.40 | -0.27 | -0.25 | -0.24 | -0.30 |
|  | 2-out-star | -0.55 | -0.68 | -0.47 | -0.52 | -0.63 | -0.40 | -0.55 | -0.42 |
|  | Isolates | -0.18 | -0.29 | -0.16 | -0.25 | -0.16 | -0.08 | -0.18 | -0.06 |
|  | AoutS(2.00) | -0.43 | -0.59 | -0.33 | -0.16 | -0.53 | -0.27 | -0.44 | -0.30 |
|  | A2p-T | -0.68 | -0.55 | -0.55 | -0.66 | -0.46 | -0.51 | -0.63 | -0.46 |
|  | Standard deviation of out-degree distribution | -0.35 | -0.39 | -0.28 | -0.36 | -0.23 | -0.20 | -0.20 | -0.26 |
|  | Skew in-degree distribution | -0.65 | -0.65 | -0.54 | -0.64 | -0.53 | -0.50 | -0.52 | -0.56 |
|  | Standard deviation of in-degree distribution | -0.55 | -0.69 | -0.45 | -0.51 | -0.63 | -0.38 | -0.54 | -0.40 |
|  | Skew out-degree distribution | -0.79 | -0.88 | -0.71 | -0.75 | -0.83 | -0.64 | -0.79 | -0.61 |
|  | Correlation between the in-degree and out-degree distribution | -0.54 | -0.38 | -0.40 | -0.53 | -0.27 | -0.34 | -0.46 | -0.29 |
|  | Global Clustering Cto | 0.23 | 0.09 | 0.23 | 0.11 | 0.34 | 0.28 | 0.39 | 0.21 |
|  | Global Clustering Cti | 0.08 | -0.14 | 0.06 | -0.04 | 0.07 | 0.11 | 0.16 | 0.04 |
|  | Global Clustering Ctm | 0.35 | 0.06 | 0.30 | 0.20 | 0.31 | 0.36 | 0.49 | 0.26 |
|  | Global Clustering Ccm | -0.34 | -0.46 | -0.31 | -0.40 | -0.31 | -0.26 | -0.24 | -0.31 |
|  | Global Clustering AKC-T | 0.46 | 0.16 | 0.41 | 0.31 | 0.42 | 0.48 | 0.60 | 0.37 |
|  | Global Clustering AKC-D | 0.37 | 0.23 | 0.36 | 0.24 | 0.49 | 0.43 | 0.54 | 0.35 |
|  | Global Clustering AKC-U | 0.17 | -0.06 | 0.16 | 0.05 | 0.17 | 0.20 | 0.26 | 0.14 |
|  | Global Clustering AKC-C | -0.31 | -0.44 | -0.28 | -0.37 | -0.29 | -0.22 | -0.20 | -0.28 |
| C | Reciprocity | 0.02 | -0.02 | 0.00 | -0.03 | -0.07 | 0.05 | -0.09 | 0.01 |
|  | Popularity | 0.00 | 0.04 | 0.00 | -0.06 | -0.02 | 0.02 | -0.09 | 0.00 |
|  | Transitivity | 0.03 | 0.01 | -0.01 | -0.06 | -0.03 | -0.01 | -0.10 | 0.02 |
|  | Absolute difference | -0.09 | -0.02 | 0.04 | 0.07 | 0.09 | 0.04 | 0.04 | 0.04 |
|  | Sender | 0.06 | -0.09 | 0.05 | 0.08 | 0.07 | 0.04 | -0.02 | 0.04 |
|  | Receiver | 0.04 | -0.10 | -0.03 | 0.00 | 0.11 | 0.06 | -0.08 | 0.01 |
|  | Age | 0.04 | 0.08 | -0.01 | -0.09 | -0.10 | 0.02 | -0.10 | 0.01 |
|  | Gender | 0.05 | -0.03 | -0.01 | -0.01 | -0.07 | 0.00 | -0.10 | 0.04 |
|  | 2-in-star | -0.24 | -0.20 | -0.25 | -0.31 | -0.35 | -0.23 | -0.35 | -0.23 |
|  | 2-out-star | -0.98 | -1.21 | -1.05 | -1.09 | -1.03 | -1.04 | -1.12 | -1.01 |
|  | Isolates | 0.56 | 0.46 | 0.46 | 0.50 | 0.44 | 0.53 | 0.40 | 0.51 |
|  | AoutS(2.00) | -0.85 | -1.10 | -0.93 | -0.98 | -0.91 | -0.01 | -1.03 | -0.86 |
|  | A2p-T | -1.03 | -1.17 | -1.13 | -1.15 | -1.09 | -1.06 | -1.21 | -1.12 |
|  | Standard deviation of out-degree distribution | -0.18 | -0.14 | -0.19 | -0.25 | -0.29 | -0.17 | -0.30 | -0.18 |
|  | Skew in-degree distribution | -0.50 | -0.46 | -0.52 | -0.59 | -0.60 | -0.49 | -0.60 | -0.49 |
|  | Standard deviation of in-degree distribution | -1.04 | -1.30 | -1.12 | -1.17 | -1.10 | -1.11 | -1.20 | -1.07 |
|  | Skew out-degree distribution | -1.53 | -1.65 | -1.55 | -1.58 | -1.56 | -1.51 | -1.61 | -1.51 |
|  | Correlation between the in-degree and out-degree distribution | -0.86 | -0.98 | -0.99 | -1.04 | -0.93 | -0.86 | -1.12 | -1.00 |
|  | Global Clustering Cto | 0.55 | 0.74 | 0.58 | 0.53 | 0.38 | 0.58 | 0.41 | 0.54 |
|  | Global Clustering Cti | -0.01 | -0.06 | -0.05 | -0.11 | -0.15 | -0.06 | -0.18 | -0.04 |
|  | Global Clustering Ctm | 0.49 | 0.56 | 0.50 | 0.44 | 0.32 | 0.46 | 0.34 | 0.48 |
|  | Global Clustering Ccm | -0.72 | -0.82 | -0.76 | -0.87 | -0.85 | -0.78 | -0.91 | -0.77 |
|  | Global Clustering AKC-T | 0.83 | 0.90 | 0.84 | 0.79 | 0.66 | 0.80 | 0.70 | 0.83 |
|  | Global Clustering AKC-D | 1.00 | 1.23 | 1.05 | 1.01 | 0.81 | 1.05 | 0.88 | 1.01 |
|  | Global Clustering AKC-U | 0.07 | 0.02 | 0.03 | -0.03 | -0.06 | 0.02 | -0.10 | 0.04 |
|  | Global Clustering AKC-C | -0.61 | -0.70 | -0.64 | -0.76 | -0.75 | -0.68 | -0.81 | -0.67 |

Note: A GOF statistic is calculated by (parameter observation-mean sample)/standard deviation.
